# An Unexpected Dependence of Cortical Depth in Shaping Neural Responsiveness and Selectivity in Mouse Visual Cortex

**DOI:** 10.1523/ENEURO.0497-19.2020

**Published:** 2020-03-17

**Authors:** Philip O’Herron, Manuel Levy, John J. Woodward, Prakash Kara

**Affiliations:** 1 Department of Neuroscience, Medical University of South Carolina, Charleston, SC 29425; 2 Department of Physiology, Augusta University, Augusta, GA 30912; 3 Department of Neurobiology, Duke University, Durham, NC 27710; 4 Department of Neuroscience, University of Minnesota, Minneapolis, MN 55455

**Keywords:** calcium, laminae, multi-photon, neocortex, selectivity, two-photon

## Abstract

Two-photon imaging studies in mouse primary visual cortex (V1) consistently report that around half of the neurons respond to oriented grating stimuli. However, in cats and primates, nearly all neurons respond to such stimuli. Here we show that mouse V1 responsiveness and selectivity strongly depends on neuronal depth. Moving from superficial layer 2 down to layer 4, the percentage of visually responsive neurons nearly doubled, ultimately reaching levels similar to what is seen in other species. Over this span, the amplitude of neuronal responses also doubled. Moreover, stimulus selectivity was also modulated, not only with depth but also with response amplitude. Specifically, we found that orientation and direction selectivity were greater in stronger responding neurons, but orientation selectivity decreased with depth whereas direction selectivity increased. Importantly, these depth-dependent trends were found not just between layer 2/3 and layer 4 but at different depths within layer 2/3 itself. Thus, neuronal depth is an important factor to consider when pooling neurons for population analyses. Furthermore, the inability to drive the majority of cells in superficial layer 2/3 of mouse V1 with grating stimuli indicates that there may be fundamental differences in the micro-circuitry and role of V1 between rodents and other mammals.

## Significance Statement

Studies frequently pool responses of neurons from different cortical depths in population analyses. Here, we show that population neuronal response characteristics in mouse primary visual cortex (V1) vary dramatically across depth planes separated by just 50 μm. We also demonstrate that the stimulus selectivity of neuronal responses varies with both cortical depth and the response amplitude of neurons. These findings highlight the importance of considering cell depth and response amplitude as important factors contributing to the overall characteristics of neurons in sensory cortex.

## Introduction

With the emergence of two-photon imaging as a tool for systems neuroscience over the past 15 years, there has been an enormous increase in the use of the mouse as a model system to study cortical neuronal physiology. Mice are easily genetically modified to label specific populations of neurons with fluorescent indicators and opsins, allowing the study of cell type-specific circuitry (Luo et al., 2008; Scanziani and Häusser, 2009; Zhao et al., 2011; Adesnik et al., 2012; Lee et al., 2012). Additionally, because they are much smaller than cats and primates, brain pulsations due to respiration and heartrate are easier to control. Although these features have made the mouse a valuable model in many respects, some differences have been noted between the visual system of mice and that of cats and primates. The functional organization of neurons into orientation maps that is prevalent across many species is absent in rodents (Ohki et al., 2005; but see Fahey et al., 2019). Additionally, the proportion of pyramidal neuronal synapses onto inhibitory neurons was found to be much greater in rodents than in cats or monkeys (Bock et al., 2011; Bopp et al., 2014).

A further difference that has become apparent in the literature is the percentage of responsive neurons in the cortex. An early electrophysiological study reported that most neurons (87%) in mouse V1 were responsive to grating stimuli (Niell and Stryker, 2008). However, electrophysiological recordings are blind to “silent” neurons which do not show spontaneous or evoked activity during the recordings, and thus the true percentage of all neurons that are responsive is difficult to measure. Subsequent studies using two-photon calcium imaging, which can record activity levels from all neurons in a region, have consistently reported that around half of the neurons in mouse V1 respond significantly to oriented grating stimuli (Mrsic-Flogel et al., 2007; Sohya et al., 2007; Kerlin et al., 2010; Smith and Häusser, 2010; Bonin et al., 2011; Ebina et al., 2014; Ayzenshtat et al., 2016; Palagina et al., 2017). However, the few two-photon calcium imaging studies done in V1 in primates and cats indicate a much higher percentage of responsive neurons to grating stimuli – typically >90% of the neurons are responding (Kara and Boyd, 2009; Nauhaus et al., 2012; Shen et al., 2012; Ikezoe et al., 2013; Li et al., 2017).

In addition to these differences in responsiveness and functional connectivity, the mouse neocortex is also much thinner than that of cats and primates. As a result, neurons from layers 1 through 4 can be easily accessed with conventional two-photon imaging techniques in the mouse visual cortex, whereas in cats and primates two-photon imaging is limited to the upper portion of layer 2/3. Although the ability to image more of the neocortex in the mouse can certainly be an advantage, it raises important considerations about how populations of neural activity should be analyzed and the functional differences of these populations across species. Layers of the neocortex have different anatomical and functional characteristics and play different roles in the basic circuitry of information processing (Douglas and Martin, 1991; D’Souza and Burkhalter, 2017). Yet, most imaging studies in mouse V1 pool neuronal responses without regard for recording depth. Even when layer is taken into consideration, (Kondo and Ohki, 2016; Sun et al., 2016; Yildirim et al., 2019) it is very rare to distinguish neurons by depth within a layer.

In the present study, we sought to determine how the response properties of neurons in mouse V1 depend on cortical depth. We show that although in superficial layer 2/3 only around half of the neurons are responding to grating stimuli, deep in layer 2/3 and in layer 4, nearly all the neurons are responsive, similar to what is seen in cats and primates. We demonstrate that the amplitude and the selectivity of neuronal responses to drifting grating stimuli depend on imaging depth – not only across laminae but even within layer 2/3 itself. Neuronal selectivity to orientation and direction across the population changes little with cortical depth, in agreement with previous studies (Niell and Stryker, 2008; Ma et al., 2010; Van den Bergh et al., 2010; Durand et al., 2016; Kondo and Ohki, 2016; Sun et al., 2016; Yildirim et al., 2019). However, this apparent homogeneity masks strong differences in orientation and direction selectivity when neurons are separated according to both depth and response strength. We discuss the implications of our findings with respect to the differences in visual processing between mice, cats and primates.

## Materials and Methods

Animals and surgery. All surgical and experimental procedures were approved by the Institutional Animal Care and Use Committee at Medical University of South Carolina (MUSC). All experiments were performed at MUSC. C57Bl/6J mice (*n *=* *7 male, postnatal day 90–111) were initially anaesthetized with a bolus infusion of fentanyl citrate (0.04–0.05 mg kg^−1^), midazolam (4–5 mg kg^−1^), and dexmedetomidine (0.20–0.25 mg kg^−1^). During two-photon imaging, continuous intraperitoneal infusion with a lower concentration mixture (fentanyl citrate: 0.002–0.003 mg kg^−1^ h^−1^, midazolam: 0.2–0.3 mg kg^−1^ h^−1^, and dexmedetomidine: 0.010–0.15 mg kg^−1^ h^−1^) was administered using a catheter connected to a syringe pump. The heart and respiration rates of the animals were continually monitored throughout the surgeries and imaging. Craniotomies (2–3 mm) were opened over the primary visual cortex (V1) centered ∼2.5 mm lateral to the lamda suture and 1–1.5 mm anterior to the transverse sinus. A pipette containing a solution with Oregon Green 488 Bapta-1 AM (OGB-1 AM) and a red dye (Alexa Fluor 633 or Alexa Fluor 594) was inserted into the craniotomy and the dye was injected with pressure puffs under continuous visual guidance using two-photon microscopy (O'Herron et al., 2012). Pipette tips were positioned between 160 and 265 μm deep for the injections (mean depth 193 μm across seven animals). After waiting 1 h, the dura was removed and the craniotomies were sealed with agarose (1.5–2% dissolved in artificial cerebrospinal fluid) and a 5-mm glass coverslip.

Fluorescence was monitored with a custom-built microscope (Prairie Technologies) coupled with a Mai Tai (Newport Spectra-Physics) mode-locked Ti:sapphire laser (810  or 920 nm) with DeepSee dispersion compensation. Excitation light was focused by a 40× (NA 0.8, Olympus) water immersion objective. Full frame imaging of ∼300-μm square windows was obtained at ∼0.8 Hz.

Drifting square-wave grating stimuli were presented to the contralateral eye on a 17-inch LCD monitor. The gratings were presented at 100% contrast, 30 cd m^−2^ mean luminance, 1.5-Hz temporal frequency, and 0.033–0.063 cycles/°. Stimuli were optimized for retinotopic position and spatial frequency preference right at the layer 1/layer 2 border (typically around 120-μm depth). Our injection site usually yielded receptive fields close to eye level (0° elevation) and roughly perpendicular to the eye (azimuth around 50–70°). At each of our depth planes, drifting gratings were presented at 16 directions of motion in 22.5° steps (except 1 of 35 runs which used 8 directions in 45° steps) for 6.5 s with 13 s of blank before each stimulus. Each condition was repeated at least 8 times except for two runs with only five repetitions due to the removal of later repetitions on account of large movements.

Images were analyzed in MATLAB (MathWorks) and ImageJ (National Institutes of Health). Data with significant movements (several micrometers) in XY or Z were excluded. Data with small drift movements were realigned by maximizing the correlation between frames. Cell masks were automatically created based on morphologic features and then subsequently refined by hand. Astrocytes were removed from the data based on morphologic criteria (Gandhi et al., 2008; Runyan et al., 2010; Van Hooser et al., 2012) and in 2 animals we verified this method by labeling astrocytes with Sulforhodamine 101. Fluorescence time courses for each cell were computed by averaging over the pixels in each mask. The time courses were corrected for neuropil contamination similar to Kerlin et al. (2010). First, out of focus neuropil contamination was estimated from the fluorescence in small vessels (<15 μm). The fluorescence from hand-drawn vessel masks was divided by the fluorescence of the surrounding neuropil to obtain an estimate of the fraction *C* of the response that is attributable to out of focus contamination. Then the fluorescence time course for soma masks were corrected by subtracting this fraction of the surrounding neuropil fluorescence. So:

(1) $${\text{F}}_{\text{cell}\_\text{true}}\left(\text{t}\right)={\;\text{F}}_{\text{cell}\_\text{measured}}\left(\text{t}\right)–C\times {\;\text{F}}_{\text{neuropil}}\left(\text{t}\right),$$

where t is time and F is fluorescence. Values for *C* were between 0.35 and 0.72 (median = 0.56). Neuropil masks were created by expanding a spherical shell 15 μm beyond the soma masks. The inner 3 μm were excluded as a buffer zone around each neuron. Pixels were also excluded from the neuropil masks if they belonged to other soma masks and their 3 μm shells, blood vessels, non-neuronal cell bodies (such as astrocytes), or neuronal somas that were too out of focus to be included in the population. The radius of the neuropil mask was expanded, if necessary, until the neuropil area was greater than 10 times the soma area. The median radius of the neuropil masks was 14 μm and the range was 12–32 μm.

The time courses for each neuron were then normalized by a sliding baseline of the mean fluorescence of the last four frames of each blank interval. The responses to each condition (ΔF/F) were computed as (F_1_-F_0_)/F_0_ where F_1_ was the average fluorescence across all 5 stimulus frames and F_0_ was the average of the last four frames of each blank interval. Neurons were defined as responsive using ANOVA across the 16 directions and the blank intervals (*p *<* *0.01). Because different studies have used different criteria for responsiveness, we did two additional analyses using different criteria: (1) with *p* set to 0.001, and (2) where responsiveness was defined as average ΔF/F > 5% for at least one stimulus direction. The data showed the same depth-dependent trends in both cases (Table 1). The response amplitude was computed for all responsive neurons.

The Orientation Selectivity Index (OSI) was defined as:

(2) $$OSI=abs(\frac{\sum_{k}r_{k}e^{i2\theta_{k}}}{\sum_{k}r_{k}}),$$

where $\theta_{k}$ is the orientation of each stimulus and $r_{k}$ is the mean response across trials to that stimulus (Swindale, 1998; Ringach et al., 2002). Note that OSI = 1 - circular variance. The Orientation Modulation Index (OMI) was defined as:

(3) $$OMI=\left(R_{pref}-R_{ortho}\right)/(R_{pref}+R_{ortho}),$$

where $R_{pref}$ is the response to the stimulus direction that evoked the strongest response and $R_{ortho}$ is the average of the responses to the two orthogonal stimuli. Note that this metric is sometimes referred to as the OSI while what we term the OSI is sometimes referred to as the global OSI (gOSI; Kondo and Ohki, 2016; Sun et al., 2016; Yildirim et al., 2019). The OSI and OMI were computed on all responsive neurons.

To analyze the tuning width of the neurons, we first screened the population for selective neurons. Neurons were tested to see if any of the stimulus conditions evoked a significantly different response from any other (ANOVA, *p *<* *0.01). If so, each neuron’s responses were then fit with a least-squares method to a Von Mises function:

(4) $$f(\theta )=A\cdot e^{\kappa (\cos(2(\theta -\varphi ))-1)}+B,$$

where θ is the orientation values, A corresponds to the peak amplitude, φ to the preferred orientation, κ is a width parameter, and B reflects the baseline response (Swindale, 1998). If the peak of the curve was not at least twice the trough (approximating the preferred response being at least twice the magnitude of the least preferred), neurons were excluded. Additionally, the R^2^ for the fit had to be at least 0.5 for inclusion in the selective population. For neurons that passed these criteria, we computed a population average response for each animal and depth plane. Each neuron’s responses were normalized to the peak response of that neuron and the preferred orientation was set to 0° before averaging.

Bandwidth was computed based on Swindale (1998):

(5) $$BW=\cos^{-1}\{\left(\ln0.5+\kappa \right)/\kappa \}.$$

This metric gives the full bandwidth of the curve in degrees and is independent of the baseline level.

To compute direction selectivity, we fit a double von Mises curve to the population average responses in each animal and depth plane:

(6) $$f(\theta )=A_{1}\cdot e^{\kappa (\cos(2(\theta -\varphi_{1}))-1)}+A_{2}\cdot e^{\kappa (\cos(2(\theta -\varphi_{2}))-1)}+B,$$

where $A_{\text{1}}$ was the amplitude at the preferred direction, $A_{\text{2}}$ was the amplitude of the second peak, and $\varphi_{2}$ was constrained to be $\varphi_{1}$ + 180°. The other parameters are as above.

The Direction Modulation Index (DMI) was defined as:

(7) $$DMI=(A_{1}-A_{2})/(A_{1}+A_{2}),$$

Stimulus-evoked “shadowing” from large surface vessel dilation can cause dimming of fluorescence from neurons during stimulus presentation windows. When neurons are not responding or responding weakly, this shadowing may lead to negative ΔF/F values from these neurons (Shen et al., 2012). This is because surface arteries in visual cortex show strong dilations to all stimulus conditions (O'Herron et al., 2016) while neurons may only fluoresce strongly to a few stimulus conditions. Including these negative responses in the computation of the OSI and OMI can lead to aberrant values. Therefore, if neurons showed decreased fluorescence to stimuli, we shifted all responses up by the most negative value (setting that value to zero). For consistency, all selectivity measures were performed on this shifted data. To ensure that this correction was not a confound, we performed two control analyses. First, we computed the tuning width without this correction and saw similar results (Table 1, uncorrected data). Second, we rectified all the negative responses to zero without adjusting the positive responses. We then checked all of the selectivity metrics and again found that, despite small differences in the values of some metrics, they all showed the same depth-dependent trends (data not shown).

## Results

**Figure 1.**
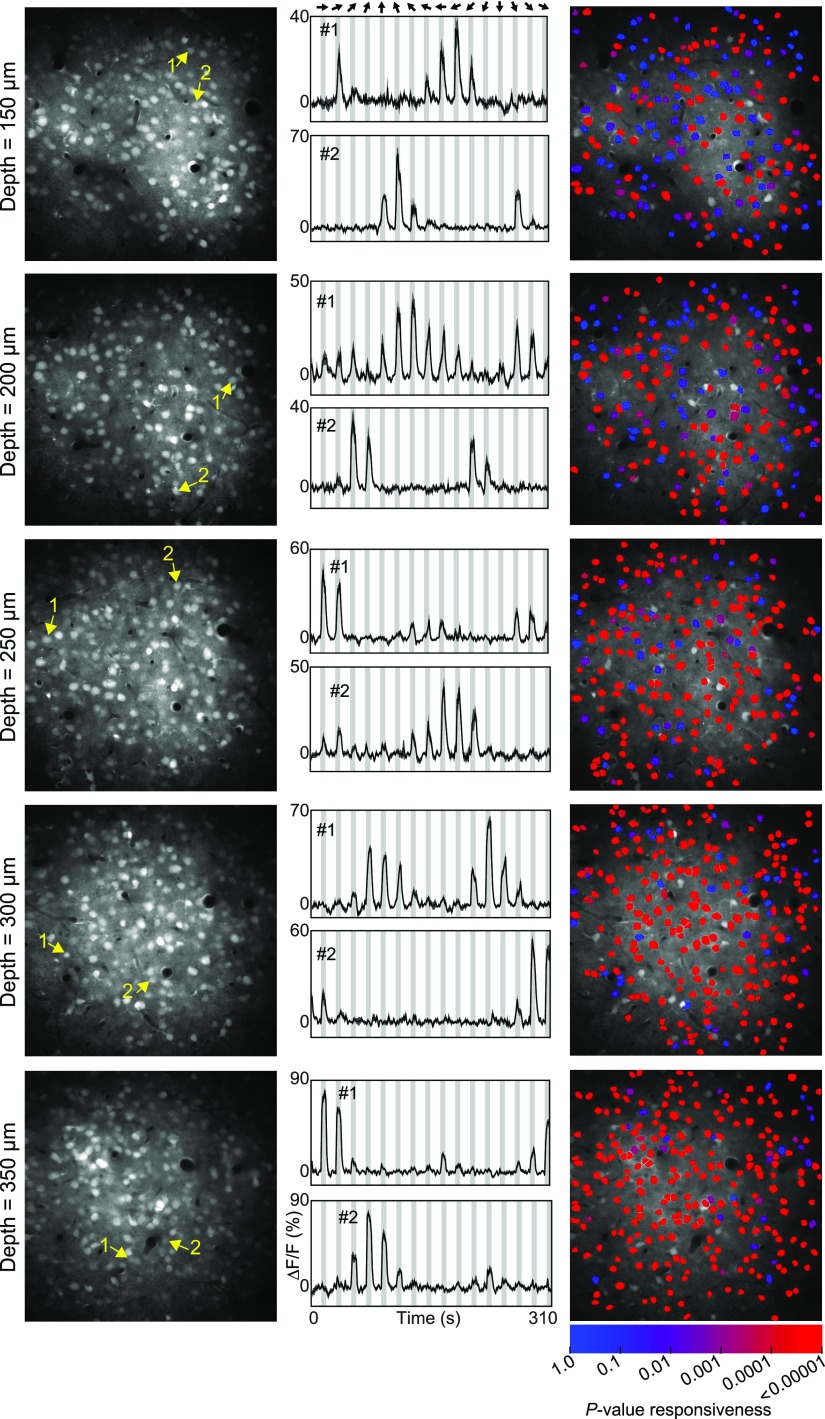
Increased neural responsiveness with cortical depth in mouse V1. Left, Anatomical images of five different depth planes from one mouse. Center, Time courses of responses from two example cells from each depth plane as indicated by yellow numbers/arrows in left column. Right, Neuronal cell masks are color coded by the *p* value from the ANOVA for responsiveness. With increasing depth there are more cell masks colored in redder hues, indicating increased responsiveness. See Extended Data Figure 1-1 for pixel-based direction maps across cortical depth. Also see Extended Data Figure 1-2 for cortical responses to pharmacological stimuli.

**Figure 2.**
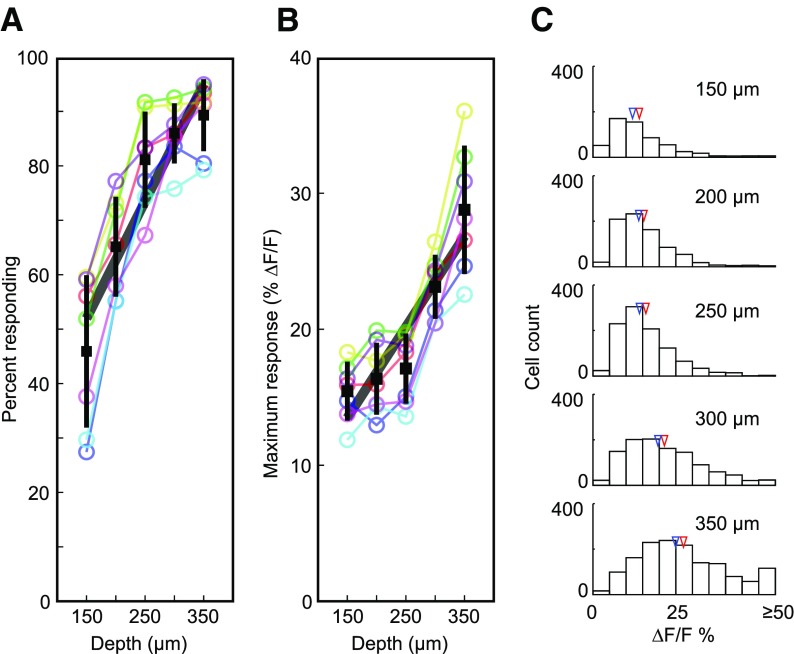
Population summary of cortical depth dependence on neural responsiveness and response amplitude. ***A***, Percentage of responding neurons as a function of imaging depth. In this and subsequent panels/figures, colored lines and circles correspond to individual mice and black squares correspond to the population average at each depth. Error bars indicate SD. The thick gray line is the linear fit to the individual runs. ***B***, Change in response amplitude with depth. ***C***, Histogram of the distribution of response amplitude across the neuronal population at each depth plane. Blue arrows correspond to the median and red arrows to the mean.

We imaged calcium responses in the mouse visual cortex at depth planes ranging from 150 μm to 350 μm below the surface in 50-μm increments. We used the synthetic calcium indicator OGB-1 AM. When injected by visualized guidance under two-photon microscopy (see Materials and Methods; Kara and Boyd, 2009; O'Herron et al., 2012; Shen et al., 2012), this dye uniformly labels all cells within a small region of tissue (300–400 μm around the injection site) regardless of lamina. This labeling strategy ensures that the responsiveness of all the neurons in each imaged plane can be determined (see Discussion). In our study, the density of neurons in mouse visual cortex was similar across depth planes except for an increase in the deepest plane (Fig. 1; Table 1). Based on the cell count and on the delineation of layers in the literature (Ji et al., 2015; Durand et al., 2016; Kondo and Ohki, 2016; Sun et al., 2016), the three superficial depths (150, 200, and 250 μm) are within layer 2/3, the 350 μm depth is within layer 4, and the 300 μm depth is near the border of layers 3 and 4. Across all depth planes we found that many neurons responded robustly to drifting grating stimuli (Fig. 1; Extended Data Fig. 1-1). However, the proportion of responsive neurons (ANOVA across all stimuli plus blank presentations; see Materials and Methods) increased dramatically with depth (Figs. 1, 2*A*; Extended Data Fig. 1-1). The average percentage of responsive neurons in superficial layer 2/3 was ∼50%, similar to what has been reported in the literature (Mrsic-Flogel et al., 2007; Sohya et al., 2007; Kerlin et al., 2010; Smith and Häusser, 2010; Bonin et al., 2011; Ebina et al., 2014; Ayzenshtat et al., 2016; Palagina et al., 2017). Deeper in layer 2/3 this ratio rose to >80%, and in layer 4 around 90% of neurons were responding. This increase in responsiveness with depth was highly significant (*R*^2^ = 0.70, *p *<* *10^−9^, linear regression, *n *=* *35 imaged regions in seven mice; Fig. 2*A*; Table 1) and was observed in every animal tested (Fig. 2*A*; Table 1; *R*^2^ > 0.79 and *p *<* *0.04 for all animals, *n *=* *5 regions per animal). Nearly all neurons (95%) within layer 2/3 responded to pharmacological stimuli (glutamate puffs, see Extended Data Fig. 1-2; Discussion), thus excluding the possibility that non-visually responsive neurons were unhealthy. Out of a total population of 6720 neurons imaged across all animals and depths, 5020 were significantly responsive to visual stimuli and were analyzed further.

10.1523/ENEURO.0497-19.2020.f1-1 Extended Data Figure 1-1 Pixel-based direction maps in mouse V1. For every mouse and imaging plane, each pixel is color coded according to the preferred stimulus direction. The hue gives the preferred direction, the saturation gives the selectivity, and the brightness gives the response strength. This analysis is independent of any cell masks but responding neurons clearly pop out as colored balls. In every mouse the density of these responsive neurons increases with deeper imaging planes through cortex. Download Figure 1-1, EPS file.

10.1523/ENEURO.0497-19.2020.f1-2 Extended Data Figure 1-2 Control experiment; 95% of cells in superficial cortical layers of mouse V1 respond robustly to exogenous glutamate application. ***A***, Representative example of an imaged plane 170 μm below the pial surface. Neuronal masks are color coded by the *p* value from the ANOVA for responsiveness to glutamate (see below). A pipette (schematized in pink) was filled with 50 mM glutamate and 100 μM Alexa Fluor 633 (for visualization of the glutamate ejection area). The pipette tip size was ∼1 μm in diameter. This glutamate pipette was positioned approximately in the center of a region of cortical layer 2/3 that was bulk-loaded with OGB-1 A.M. The dashed ring shows the 100-μm diameter region around the pipette tip. Glutamate was locally injected into the cortical tissue four times, each puff of glutamate was applied for three seconds with 22 s between the puffs (each puff 10–20 psi). In this example, 100% of the neurons in the 100-μm diameter region around the pipette tip were activated by glutamate. Further away from the tip where the glutamate did not reach, most of the neurons were unresponsive. Across four regions in three animals (depth range: 170–225 μm; mean: 200 μm) 95% of the neurons in a 100-μm diameter window around the pipette tip were significantly responsive (ANOVA; baseline imaging frames vs glutamate puff imaging frames, *p *<* *0.01). Scale bar, 100 μm. ***B***, Example responses to the glutamate injections from two cells labelled in ***A*** with yellow numbers/arrows. The blue traces are the individual repetitions and the black trace is the average. The vertical gray band indicates the glutamate injection period. Download Figure 1-2, EPS file.

The depth dependence of neuronal responsivity to visual stimuli was observed not only in the proportion of responsive neurons but also in their maximum response amplitude. Responses to the preferred stimulus direction nearly doubled from 15.5 ± 2.2% (mean ΔF/F ± SD across runs) in superficial layer 2/3 to 28.9 ± 4.7% in layer 4 (Fig. 2*B*,*C*). This depth dependence of preferred response amplitude was highly significant (*R*^2^ = 0.66, *p *<* *10^−8^, *n *=* *35 regions neurons from seven animals; Fig. 2*B*; Table 1).

**Figure 3.**
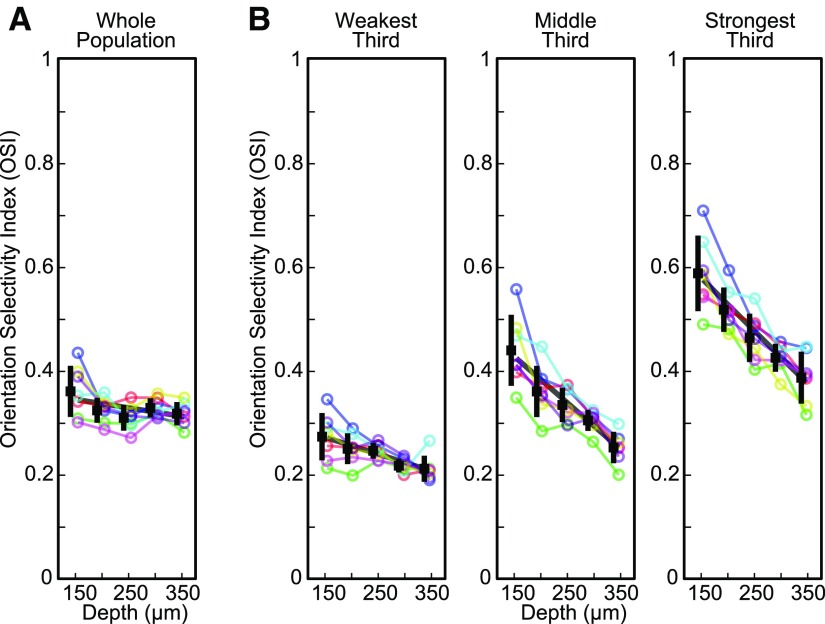
Cortical depth dependence of the OSI. ***A***, Average of OSI values for each mouse and imaging depth. ***B***, Average after dividing neurons into three groups based on response amplitude. Conventions as in previous figure. Also see Extended Data Figure 3-1 for the OSI of every responsive neuron in the population grouped by response amplitude and depth.

Previous studies reported that orientation selectivity remains approximately constant across cortical depth (Niell and Stryker, 2008; Ma et al., 2010; Van den Bergh et al., 2010; Durand et al., 2016; Kondo and Ohki, 2016; Sun et al., 2016; Yildirim et al., 2019). However, as we showed above, neuronal responsiveness changes with cortical depth. The effects of these two factors (response strength and cortical depth) on orientation selectivity have not been considered separately in previous studies. We computed the OSI (see Materials and Methods), a fit-free index that quantifies the spread of neuronal responses across orientations (OSI = 1 when a neuron responds only to one orientation). When the OSI was pooled across all neurons at each depth plane, we found that orientation selectivity very slightly decreased with depth, going from 0.36 ± 0.05 in superficial layer 2/3 to 0.32 ± 0.02 in layer 4 (*R*^2^ = 0.12, *p *=* *0.04, *n *=* *35 regions neurons from seven animals; Fig. 3*A*; Table 1). However, a more detailed analysis of the effects of depth and responsivity on OSI showed these two factors affected orientation selectivity in different ways. When neurons were divided into three equal-sized groups based on their preferred response amplitude (weak responders, ΔF/F < 15%, *n* = 1672; middle responders 15% ≤ ΔF/F ≤ 24%, *n* = 1673; strong responders, ΔF/F > 24%, *n* = 1675), for each group, neurons were much less orientation selective deeper in the cortex (Fig. 3*B*). The change in OSI with depth was particularly pronounced for strong responders (*R*^2^ = 0.69, *p *<* *10^−9^) and middle responders (*R*^2^ = 0.67, *p *<* *10^−8^) compared with weak responders (*R*^2^ = 0.41, *p *<* *10^−4^). Additionally, independent of depth, strong responders were more orientation selective than middle responders (one-way ANOVA with response group as factor, Tukey’s *post hoc* test *p *<* *10^−7^) which, in turn, were more selective than weak responders (*p *<* *10^−9^). Thus, orientation selectivity is affected by two competing trends. On the one hand, for a given level of responsiveness, orientation selectivity decreases when depth increases. On the other hand, at a given depth, orientation selectivity increases when neuronal responses increase. The two trends cancel each other out because depth covaries with neuronal responsivity (Extended Data Fig. 3-1), resulting in approximately constant OSI across depth.

10.1523/ENEURO.0497-19.2020.f3-1 Extended Data Figure 3-1 The OSI of every responsive neuron in the population grouped by response amplitude and depth. Colors indicate response amplitude group. Depth positions were randomly scattered around the true depths for better visualization. Squares indicate population average values with the size indicating the number of neurons in each population. Although all response groups showed decreased OSI with depth, superficially there are more weak responders with low OSI and deeper there are more strong responders with high OSI so the total population OSI at each depth is relatively unchanged. Download Figure 3-1, EPS file.

**Figure 4.**
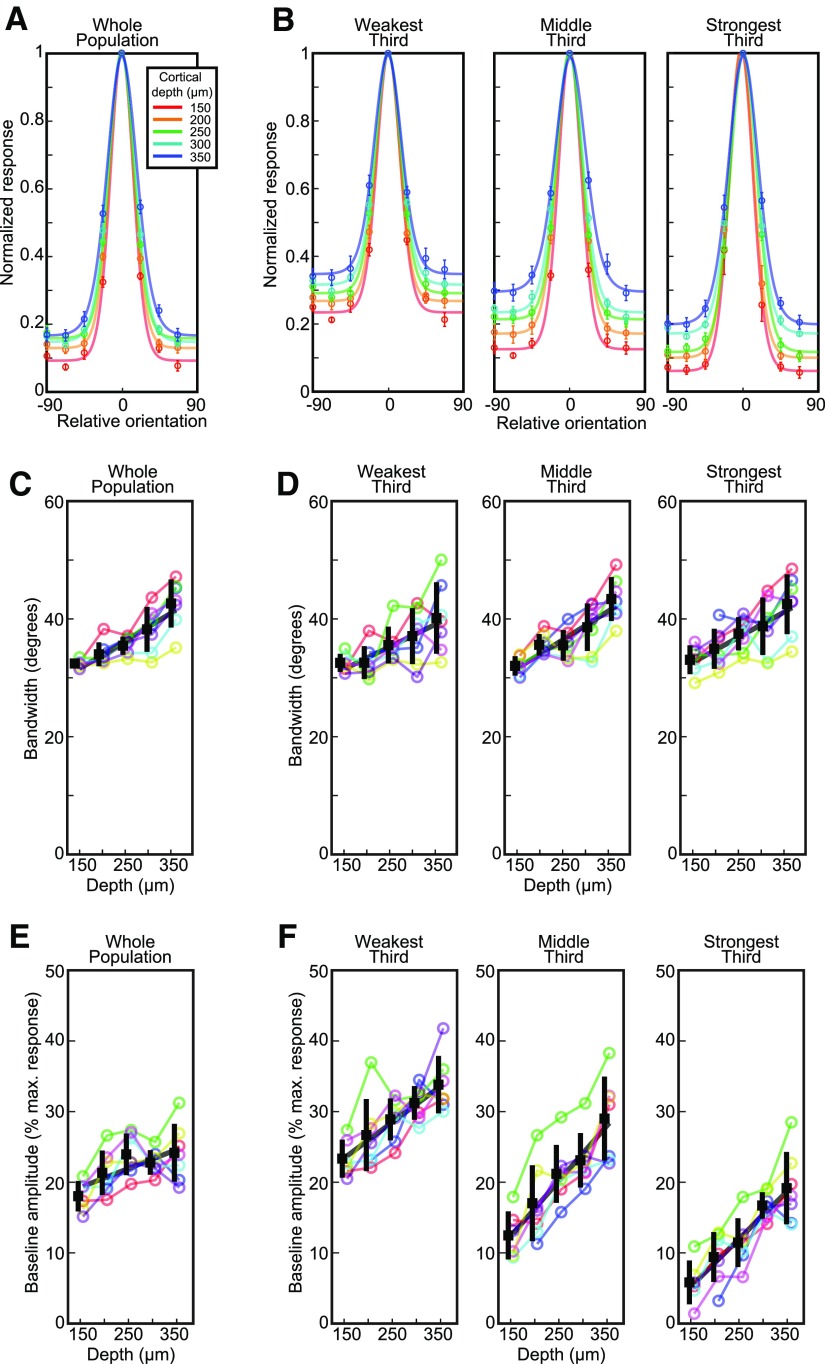
Cortical depth dependence of orientation tuning width. ***A***, At each imaging depth, the population average response is shown and fit with a tuning curve. Averages were computed for each run (five depth planes, seven animals) after aligning preferred orientations and normalizing to the maximum response for each neuron. The responses at each depth plane were then averaged across animals to obtain the population average (circles) and SD (error bars). ***B***, Population averages grouped by response amplitude. ***C***, The bandwidth of the tuning curves for each animal/depth plane (colored circles) and the population average (black squares) and SD (black bars). The gray line is the linear fit to the individual animal data. ***D***, Similar to panel ***C*** but for neurons grouped by response amplitude. ***E***, ***F***, Similar to ***C***, ***D*** but for the baseline amplitude of the tuning curves.

In order to understand in more detail how depth affects orientation tuning, we fit the responses with a von Mises curve (Swindale, 1998). First, responsive neurons were screened for orientation selectivity (see Materials and Methods) which excluded ∼24% of the responsive neurons (1187/5020). The responses of the remaining 3833 orientation selective neurons were normalized and aligned relative to each neuron’s preferred stimulus orientation, and a von Mises curve was fit to the population average of each animal at each depth (Fig. 4*A*). We found that the two parameters in these fitted curves, the bandwidth (Eqs. 4, 5) and the baseline (parameter B in Eq. 4), both increased from superficial layer 2/3 to layer 4 (bandwidth: 32.4° in superficial layer 2, 42.6° in layer 4, *R*^2^ = 0.62, *p *<* *10^−7^; baseline: 18% of peak response in layer 2, 24% in layer 4, *R*^2^ = 0.28, *p *<* *0.005; Fig. 4*A*,*C*,*E*; Table 1). Note that parameter B in Equation 4 does not represent spontaneous activity but is the weakest of all visually-evoked responses to the various oriented stimuli presented.

We separately analyzed the effects of response strength and depth on orientation tuning curves by dividing the selective neurons into three equal groups based on response amplitude (weak responders, ΔF/F < 16%, *n* = 1277 neurons; middle responders 16% ≤ ΔF/F ≤26%, *n* = 1277 neurons; strong responders, ΔF/F > 26%, *n* = 1279 neurons). We observed the same trends as for the OSI: namely that stronger responders tended to be more orientation selective than weak responders, and that their tuning curves varied more with depth (Fig. 4*B*). The fits showed that although bandwidth increased with depth in each response amplitude group, it did not vary across response groups (depth factor *p *<* *10^−10^; response group factor *p *=* *0.12; two-way ANOVA; Fig. 4*B*,*D*; Table 1). In contrast, baseline depended strongly on both depth and response strength: deep neurons and weakly responding neurons showed high baseline responses at all orientations, whereas neurons that were located more superficially and strong responders had lower baselines and were consequently more orientation selective (depth factor *p *<* *10^−16^; response group factor *p *<* *10^−27^; Fig. 4*B*,*F*; Table 1).

**Figure 5.**
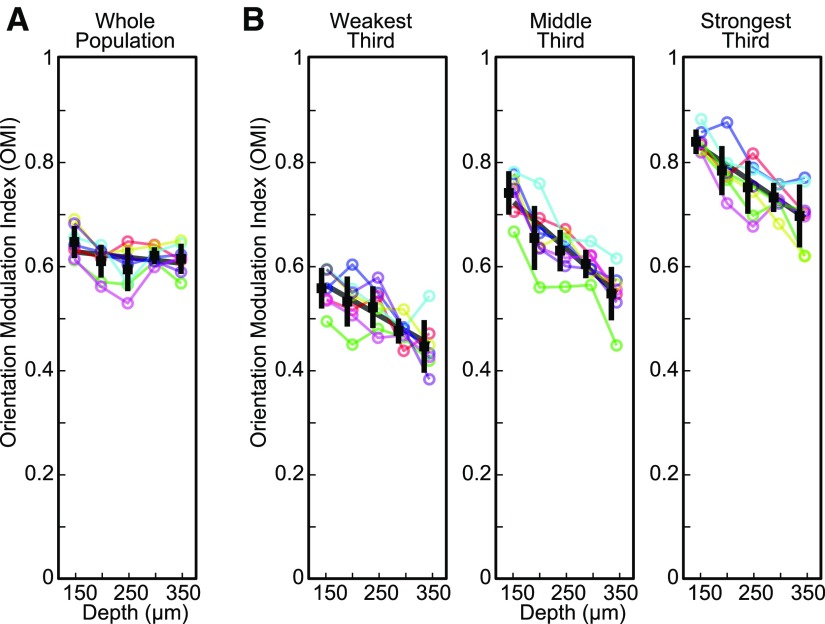
Cortical depth dependence of the OMI. ***A***, Average of OMI values for each mouse and imaging depth. ***B***, Average grouped by neuronal response amplitude. Conventions as in previous figures.

Another metric often used to quantify orientation selectivity is the difference in response between the preferred and orthogonal stimuli divided by their sum (but see Mazurek et al., 2014 for its limitations). We computed this OMI (see Materials and Methods) for each of the responsive neurons. The results were similar to what we found for the OSI except that the OMI values were consistently higher than the OSI values. The whole population showed essentially no change in selectivity with depth (*R*^2^ = 0.05, *p *=* *0.18, regression; ANOVA, *p *=* *0.05; Fig. 5*A*; Table 1). However, the separate groups all showed significant decreases in OMI with depth (Fig. 5*B*; Table 1) and the stronger responding neurons had greater OMI values (one-way ANOVA with response group as factor, Tukey’s *post hoc* test *p *<* *10^−9^ for both strongest vs middle and middle vs weakest; Fig. 5*B*).

**Figure 6.**
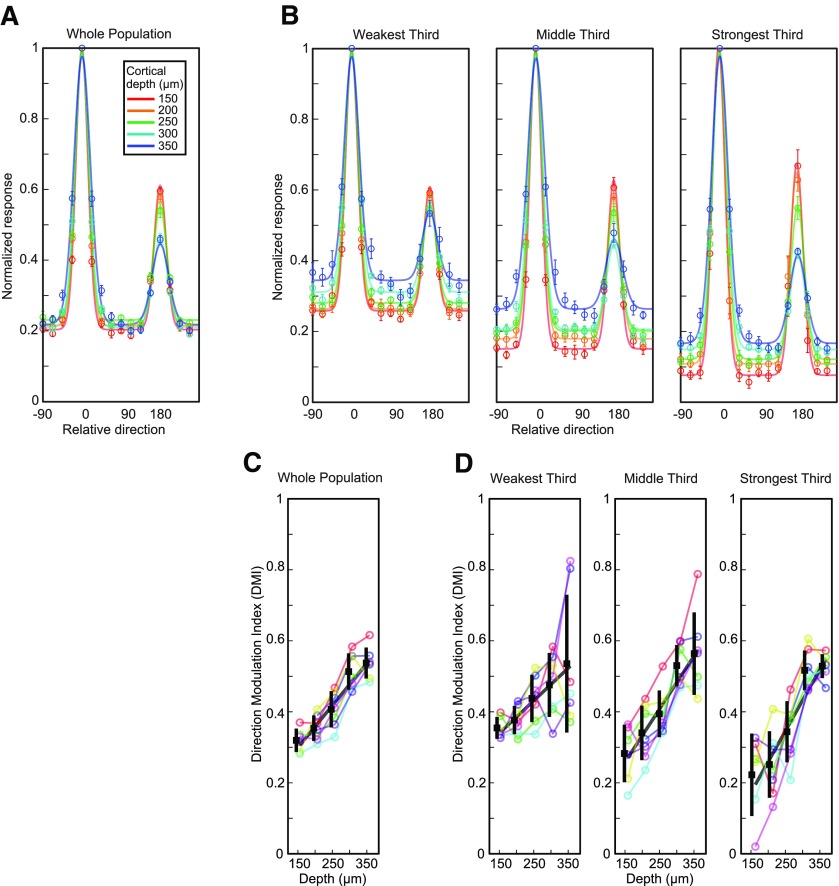
Cortical depth dependence of direction selectivity. ***A***, Population average responses across all 16 directions at each depth were fit with a dual peak tuning curve (one peak for each of the two orthogonal directions; see Materials and Methods). ***B***, Same as *A*, but for populations grouped by response amplitude. ***C***, DMI computed from the fits. ***D***, Same as ***C*** but for neurons grouped by response amplitude. Conventions as in Figure 4.

Finally, the data were analyzed to determine the effect of depth on direction selectivity. Surprisingly, we found that direction selectivity increased with cortical depth, and thus followed a pattern opposite to orientation selectivity. We computed population tuning curves for each depth as above but without averaging across directions for each orientation. The superficial neurons had a larger response to the null direction indicating reduced direction selectivity compared with the deeper neurons (Fig. 6*A*). The DMI (see Materials and Methods) increased with depth, from 0.32 ± 0.03 in superficial layer 2/3 to 0.54 ± 0.04 in layer 4 (*R*^2^ = 0.78, *p *<* *10^−9^; Fig. 6*C*; Table 1). When we separated neurons by response amplitude, we found that the DMI increased with depth across all response groups and the effect of depth was more pronounced with stronger responding neurons (Fig. 6*B*,*D*; Table 1). In the strongest responding group, the DMI nearly tripled from 0.22 in superficial layer 2/3 to 0.53 in layer 4 (Fig. 6*D*; Table 1).

## Discussion

Our findings demonstrate that neuronal responses in mouse V1 strongly depend on cortical depth. The percentage of responsive neurons and the amplitude of the responses increase by a factor of 2 with imaging depth from upper layer 2/3 into layer 4. By specifically considering the effect of cortical depth independent from changes in responsiveness, we showed that deeper neurons are less orientation selective than superficial neurons, largely due to increased unspecific responses. On the contrary, direction selectivity increased with cortical depth. Depth dependent changes did not occur only at the border between layer 3 and 4, but also across planes (150, 200, and 250 μm) located within layer 2/3.

### Determining the responsiveness of the visual cortex

To accurately determine the percentage of responsive neurons in a region of cortex, one needs to monitor all the neurons in the tissue without bias. Electrophysiological recordings cannot achieve this since electrodes will only pick up neurons that fire action potentials and only in the region around the tip of the electrode. This limitation has led to the argument that most of V1 may be unresponsive to simple stimuli like oriented bars and gratings (Olshausen and Field, 2005; Shoham et al., 2006). In vivo two-photon microscopy bypasses this limitation since even silent neurons can be imaged. Recent two-photon imaging studies have demonstrated that nearly all of the neurons in cat and primate V1 respond to simple oriented stimuli (Kara and Boyd, 2009; Nauhaus et al., 2012; Shen et al., 2012; Ikezoe et al., 2013; Li et al., 2017). Studies to date using mice have typically reported that only about half of mouse V1 neurons respond to oriented bars and gratings (Mrsic-Flogel et al., 2007; Sohya et al., 2007; Kerlin et al., 2010; Smith and Häusser, 2010; Bonin et al., 2011; Ebina et al., 2014; Ayzenshtat et al., 2016; Palagina et al., 2017). However, these studies did not report the fraction of responsive neurons at different cortical depths. Here, we show that deeper in layer 2/3 and layer 4, nearly all the neurons in mouse V1 respond to oriented gratings.

Use of the synthetic dye OGB-AM was crucial to our study, because it labels all cells within a 300- to 400-μm diameter volume, including those that are unresponsive to visual stimuli. In contrast, genetically encoded calcium indicators, e.g., GCaMP6, can have heterogeneous expression levels in a local region of tissue (Tian et al., 2009; Chen et al., 2013; Dana et al., 2014; Wilson et al., 2017), and their low baseline fluorescence can make the detection of inactive neurons difficult (Chen et al., 2013). Furthermore, over-expression of the indicators can impair neuronal health and reduce the ability to accurately detect activity. In the present study, we found that nearly all the cells in layer 2/3 responded to local application of glutamate, indicating that they were healthy and additionally that we are able to detect their activity when present (Extended Data Fig. 1-2). This is consistent with previous studies, where the same protocol applied in the cat visual cortex (visualized dye injection at ∼200 μm below the cortical surface; see Materials and Methods) yielded healthy responses in >90% of imaged neurons, even in superficial layers (Kara and Boyd, 2009; Shen et al., 2012). It should be noted also that visually responsive and unresponsive cells were evenly spread throughout the imaged regions (Fig. 1; Extended Data Fig. 1-1) and that we did not observe clusters of unhealthy or saturated neurons even at the center of the injection. Taken together, these observations suggest that superficial neurons responded poorly to visual gratings not because they were unhealthy but rather because they perform a different function from deeper neurons.

Prior studies comparing the amplitude of responses between layer 2/3 and layer 4 of mouse V1 have typically reported few differences. One electrophysiological study found that in awake mice, evoked firing rates were higher in layer 4 than in layer 2/3 (Dadarlat and Stryker, 2017). However, this same group found no difference in mice anesthetized with urethane and chlorprothixene (Niell and Stryker, 2008). Other studies have found no difference in evoked firing between layer 2/3 and layer 4 in awake mice (Durand et al., 2016) or mice anesthetized with urethane (Van den Bergh et al., 2010; Durand et al., 2016). In contrast, in mice anesthetized with the fentanyl cocktail, we find a dramatic increase in stimulus-evoked firing rates from layer 2/3 into layer 4.

### Orientation and direction selectivity across depth

Although previous electrophysiological and imaging studies in mice have reported little variation in orientation selectivity with depth (Niell and Stryker, 2008; Ma et al., 2010; Van den Bergh et al., 2010; Durand et al., 2016; Kondo and Ohki, 2016; Sun et al., 2016; Yildirim et al., 2019), a trend toward greater orientation selectivity in more superficial layers was apparent across these studies. Our data show the same weak trend when all neurons in each depth plane are pooled together. However, when neurons were grouped by response strength, several interesting interactions between response amplitude, cortical depth, and orientation selectivity emerged. First, neurons with greater response amplitude, which are found in greater proportion deeper in layer 2/3 and layer 4, tend to have greater selectivity. Second, for a given response strength, neurons deeper in the cortex tend to have less selectivity. In other words, superficial neuronal populations mostly consisted of weakly responsive, poorly orientation selective neurons, with a few strongly responsive, highly orientation selective neurons. Deeper in the cortex, neurons were more responsive, but were also less orientation selective than more superficial neurons with comparable response strength (Extended Data Fig. 3-1). As a result, orientation selectivity at the population level remained mostly constant across depth, although different circuit mechanisms may be involved. Additionally, we found that although superficial neurons are more selective for stimulus orientation, they are less selective for stimulus direction. Increased direction selectivity in layer 4 versus layer 2/3 has been reported in one study (Sun et al., 2016) but others found no difference (Kondo and Ohki, 2016; Yildirim et al., 2019) or the opposite trend (Van den Bergh et al., 2010). Similar to orientation selectivity, grouping neurons by response strength showed that stronger responders were more direction selective and they showed greater increases in selectivity with depth than weaker responders. Interestingly, prior studies in non-rodent species have shown depth-dependent differences in orientation selectivity. In both macaques (Ringach et al., 2002) and tree shrews (Van Hooser et al., 2013) for instance, orientation selectivity was greater in superficial layer 2/3 than in deeper layer 2/3. However, in these earlier studies, layer 4 showed a similar level of selectivity to superficial layer 2/3, unlike the continuous reduction in selectivity across cortical depth that we see in mice.

### Circuits for depth dependence of response properties

One possible explanation for the increase in responsiveness with increasing depth in the mouse visual cortex may be the distribution of thalamic inputs. Genicular inputs are not as tightly constrained to layer 4 in mouse V1 (Nakamura et al., 2007; Ji et al., 2015; Kondo and Ohki, 2016; Sun et al., 2016) as they are in cats and primates (Gilbert and Wiesel, 1979; Friedlander and Martin, 1989). Rather, these inputs spread up into layer 2/3, innervating the deeper part of layer 2/3 quite strongly and becoming sparser more superficially. Thus, as the number of thalamic inputs increases with depth, the feed-forward neuronal drive could also increase, leading to a higher percentage of responding neurons and a greater response amplitude. This difference in thalamic inputs could also potentially explain the weakening of orientation selectivity with increasing depth. Because thalamic inputs to visual cortex have lower orientation selectivity than cortical neurons (Kondo and Ohki, 2016; Sun et al., 2016), the greater contribution of thalamic drive to neurons deeper in layer 3 and layer 4 could broaden the orientation tuning of these neurons relative to the more superficial neurons that receive a greater proportion of cortical inputs. It should be noted also that in the case of weakly responding neurons, orientation selectivity is inherently more difficult to measure due to the lower signal to noise ratio. This could have potentially reduced the relationship between depth and orientation selectivity we measured in this population. However, because the interaction between depth and selectivity was most pronounced in the strongest responding neurons, this rules out the possibility that the change in selectivity with depth is an artifact of analyzing noisy data.

In addition to the laminar distribution of thalamic boutons in visual cortex, the *selectivity* of the boutons themselves could also play a role in setting the selectivity for orientation and direction across layers. One study reported that geniculate boutons in layer 4 have lower orientation selectivity but greater direction selectivity than boutons in layer 2/3 (Sun et al., 2016). However, these differences are relatively small compared with the differences we see in the neurons of V1, likely reflecting intra-cortical connections that further amplify small differences seen in the boutons. For instance, neurons in layer 2/3 have been shown to be more likely to connect to neurons with the same preferred orientation, but there is little increase in connection probability for neurons with the same preferred direction (Ko et al., 2011). Thus, mouse V1 may adopt a coding strategy that favors maximizing orientation information at the expense of direction coding as information moves from layer 4 to layer 2/3. One possible reason for this coding strategy might be the smaller number of neurons in V1 of mice relative to larger mammals. It could also be the case that in mouse V1, direction information is propagated to higher areas specialized for motion and spatial processing (Wang et al., 2011; Glickfeld and Olsen, 2017) by a relatively small dedicated set of neurons in layer 2/3.

Another possibility is that the full screen gratings we used caused greater surround suppression in the superficial depths compared with layers 3 and 4. Although past studies have reported that surround suppression is weaker in the infragranular layers than in layer 2/3 or layer 4 (Nienborg et al., 2013; Vaiceliunaite et al., 2013; Self et al., 2014; Plomp et al., 2019), none reported significant differences between layer 2/3 and layer 4 or between superficial and deeper layer 2/3 (but see Van den Bergh et al., 2010).

### Implications for cortical coding

Although weaker thalamic drive may explain the smaller percentage of responding neurons in superficial layer 2/3, there is still the question of what stimuli might activate the silent half of the neurons. Previous studies have found neurons in mouse V1 that are unresponsive to single gratings but do respond to two overlapping gratings (Juavinett and Callaway, 2015; Muir et al., 2017; Palagina et al., 2017) or to contrast-noise stimuli (Gandhi et al., 2008; Niell and Stryker, 2008). Additionally, inputs to V1 from other sensory domains, which are essentially non-existent in species like cat and primate, are prevalent in the mouse (Meredith and Lomber, 2017) and so some of the silent neurons may be selective for multisensory inputs. Locomotion has been shown to dramatically increase firing rates of neurons in mouse V1 (Niell and Stryker, 2010). Although it is not clear if locomotion increased the percentage of responsive neurons, it is likely that the increase in response amplitude across the population would lead to more neurons appearing significantly responsive. Neurons have also been found in mouse V1 that respond to stimuli in the ultra-violet range but not in the visible spectrum (Tan et al., 2015) which again may account for some of the silent neurons we see here.

The depth dependence of stimulus selectivity we have found may have important implications for how information is encoded in mouse visual cortex. Theories of sparse coding have proposed that neurons in higher areas respond more sparsely than neurons in lower areas because they become selective for increasingly complex stimulus features (Barlow, 1972; Olshausen and Field, 2004). Because the mouse visual system is simpler than that of species like cats and primates, functions typically performed by higher areas in those species may be delegated to V1 in the mouse (Niell and Stryker, 2010; Gavornik and Bear, 2014; Laramée and Boire, 2015). For instance, neurons in mouse V1 have been shown to be selective to the pattern motion of a plaid stimulus and not just the motion of the individual components (Muir et al., 2015; Palagina et al., 2017) – a property generally associated with higher visual areas in cats and primates (Gizzi et al., 1990; Albright and Stoner, 1995). Additionally, a recent study has shown that many neurons in mouse V1 are more strongly driven by complex stimuli with features such as corners, curves and textures than by the traditional gabor-type stimuli that are commonly thought to match the receptive field structure of V1 neurons (Walker et al., 2019). So perhaps the unresponsive neurons we see in superficial layer 2/3 would respond to more complex stimuli that would drive neurons in higher areas of other species.

Functional properties may vary across cells located at different depths within layer 2/3 in other sensory systems as well. Studies in the somatosensory and auditory cortices of mice have described differences in layer 2 and layer 3 in terms of their response properties as well as in their neuronal cell types and connectivity (Bureau et al., 2006; Winkowski and Kanold, 2013; Staiger et al., 2015; Meng et al., 2017). This suggests that there may be a common principle of systematic differences between layer 2 and layer 3 across the primary sensory cortices in mice and that the common practice of lumping these layers together may be problematic in certain cases. It is also possible that the difference in information encoded by layers 2 and 3 may be generalizable to other species. A recent study in macaque V1 reported that, although nearly all neurons in superficial L2/3 responded to oriented bars, many of them displayed much stronger responses to more complex stimuli (Tang et al., 2018). Thus, the greater sensitivity of superficial neurons to complex stimuli may be a general property of layer 2/3 across species. In order to determine the coding strategies of the neocortex, it is critical that future studies account for the laminar location and the depth within laminae of neurons when analyzing response properties.

**Table 1:** Summary of Data

	**150 μm**	**200 μm**	**250 μm**	**300 μm**	**350 μm**	**R^2^**	***P*** **(linear regression)**	***P* (ANOVA,** **Depth factor)**
No. of cells								
Population average	181 ± 49	176 ± 36	179 ± 31	186 ± 42	237 ± 68			
Population total	1269	1233	1256	1301	1661			
Responding, %								
Mouse 1	27	55	77	84	80	0.80	0.0386	
Mouse 2	56	66	83	86	93	0.94	0.0059	
Mouse 3	52	72	92	93	94	0.82	0.0339	
Mouse 4	30	55	74	76	79	0.83	0.0311	
Mouse 5	38	58	67	85	91	0.97	0.0020	
Mouse 6	59	77	83	88	95	0.91	0.0106	
Mouse 7	60	73	91	91	92	0.80	0.0053	
Population average	46 ± 14	65 ± 9	81 ± 9	86 ± 6	89 ± 7	0.70	4.88E-10	
Δ*F*/*F*, %								
Population average	15.5 ± 2.2	16.4 ± 2.6	17.1 ± 2.6	23.1 ± 2.4	28.9 ± 4.7	0.66	2.68E-09	
Selective of responding, %								
Population average	79 ± 6	78 ± 4	76 ± 4	78 ± 5	73 ± 7	0.10	0.07	
No. of cells (responsive)								
Weak responders	317	393	481	301	180			
Moderate responders	182	285	370	362	474			
Strong responders	78	125	170	462	840			
No. of cells (selective)								
Weak responders	249	316	368	222	122			
Moderate responders	151	216	287	291	332			
Strong responders	62	92	124	363	638			
OSI								
Weak responders	0.27 ± 0.05	0.25 ± 0.03	0.25 ± 0.02	0.22 ± 0.01	0.21 ± 0.03	0.41	3.60E-05	0.0016
Moderate responders	0.44 ± 0.07	0.36 ± 0.05	0.34 ± 0.03	0.31 ± 0.02	0.25 ± 0.03	0.67	1.55E-09	1.48E-07
Strong responders	0.59 ± 0.07	0.52 ± 0.04	0.46 ± 0.05	0.43 ± 0.03	0.39 ± 0.05	0.69	7.95E-10	1.66E-07
Population average	0.36 ± 0.05	0.33 ± 0.02	0.31 ± 0.02	0.33 ± 0.02	0.32 ± 0.02	0.12	0.0377	0.0332
OMI								
Weak responders	0.56 ± 0.04	0.53 ± 0.05	0.52 ± 0.04	0.48 ± 0.02	0.45 ± 0.05	0.51	1.57E-06	1.22E-04
Moderate responders	0.74 ± 0.04	0.66 ± 0.06	0.63 ± 0.04	0.61 ± 0.03	0.55 ± 0.05	0.65	4.12E-09	2.32E-07
Strong responders	0.84 ± 0.02	0.79 ± 0.05	0.75 ± 0.05	0.73 ± 0.03	0.70 ± 0.06	0.57	1.94E-07	1.96E-05
Population average	0.64 ± 0.03	0.61 ± 0.03	0.60 ± 0.04	0.62 ± 0.02	0.62 ± 0.03	0.05	0.1838	0.0500
Tuning width, bandwidth degrees								
Weak responders	32.5 ± 2	32.6 ± 3	35.6 ± 3	37.1 ± 5	40.2 ± 6	0.35	0.0002	0.0075
Moderate responders	32.0 ± 2	35.6 ± 2	35.5 ± 3	38.6 ± 4	43.4 ± 4	0.59	9.72E-08	2.33E-06
Strong responders	33.1 ± 2	34.9 ± 3	37.5 ± 3	38.8 ± 5	42.5 ± 5	0.43	2.60E-05	1.60E-03
Population average	32.4 ± 0.7	34.0 ± 2	35.5 ± 2	38.2 ± 4	42.6 ± 4	0.62	3.99E-08	2.11E-06
Baseline response level, % max resp								
Weak responders	23 ± 3	27 ± 5	29 ± 3	31 ± 2	34 ± 4	0.53	1.18E-06	1.53E-04
Moderate responders	12 ± 3	17 ± 5	21 ± 4	23 ± 4	29 ± 6	0.61	5.96E-08	8.67E-06
Strong responders	6 ± 3	9 ± 4	11 ± 3	17 ± 2	19 ± 5	0.66	4.39E-09	8.74E-07
Population average	18 ± 2	21 ± 3	24 ± 3	23 ± 2	24 ± 4	0.28	0.0012	0.0054
DMI								
Weak responders	0.35 ± 0.03	0.38 ± 0.04	0.44 ± 0.07	0.48 ± 0.09	0.54 ± 0.19	0.31	6.47E-04	0.0234
Moderate responders	0.28 ± 0.08	0.34 ± 0.08	0.39 ± 0.07	0.53 ± 0.06	0.56 ± 0.12	0.64	1.26E-08	1.29E-06
Strong responders	0.22 ± 0.12	0.25 ± 0.09	0.34 ± 0.09	0.52 ± 0.06	0.53 ± 0.03	0.69	1.22E-09	2.70E-08
Population average	0.32 ± 0.03	0.35 ± 0.04	0.41 ± 0.05	0.51 ± 0.05	0.54 ± 0.04	0.78	4.47E-12	2.97E-10
Responding, %; *p* ≤ 0.001								
Population average	38 ± 13	57 ± 9	73 ± 11	81 ± 8	85 ± 8	0.73	8.59E-11	
Responding, %; Δ*F*/*F* ≥ 5%								
Population average	57 ± 16	79 ± 11	88 ± 6	90 ± 4	93 ± 6	0.55	3.34E-07	
Uncorrected data, tuning width								
Weak responders	30.7 ± 2	31.9 ± 3	34.7 ± 3	35.0 ± 4	36.2 ± 4	0.27	0.0016	0.0349
Moderate responders	32.1 ± 2	35.0 ± 3	34.5 ± 2	38.2 ± 4	41.2 ± 4	0.52	1.58E-06	4.60E-05
Strong responders	32.9 ± 3	34.7 ± 3	37.3 ± 3	38.0 ± 5	42.0 ± 5	0.41	4.31E-05	0.0021
Population average	31.4 ± 0.9	33.5 ± 2	34.7 ± 1	37.3 ± 4	41.1 ± 4	0.60	8.77E-08	7.36E-06
Uncorrected data, baseline response level								
Weak responders	16 ± 4	21 ± 6	24 ± 4	24 ± 4	18 ± 4	0.02	0.39	0.0127
Moderate responders	10 ± 3	17 ± 6	19 ± 6	21 ± 4	27 ± 7	0.53	9.11E-07	5.25E-05
Strong responders	4 ± 3	8 ± 4	12 ± 3	15 ± 2	19 ± 6	0.67	2.92E-09	9.74E-07
Population average	13 ± 3	18 ± 4	21 ± 4	20 ± 2	21 ± 5	0.30	7.61E-04	0.0034

For the percent responding, data for individual mice are shown in addition to the population average. The columns to the right of the depth columns give the R^2^ and *P* values for linear regression on the data and the *P* value for one-way analysis of variance with depth as the factor where applicable.

## Synthesis

Reviewing Editor: Siegrid Löwel, University of Goettingen Decisions are customarily a result of the Reviewing Editor and the peer reviewers coming together and discussing their recommendations until a consensus is reached. When revisions are invited, a fact-based synthesis statement explaining their decision and outlining what is needed to prepare a revision will be listed below. The following reviewer(s) agreed to reveal their identity: Stephen Van Hooser, Matthew Self.

This study carefully examines a depth dependence of responsiveness and tuning strength (i.e. orientation/direction selectivity) in mouse V1, which is slightly apparent in existing data sets but has never been noted before. While these novel data are not spectacular, they are robust and useful and enhance our understanding of mouse V1 responses.

The authors report calcium signals from anesthetized mouse V1 in response to drifting grating stimuli. While this is a commonly used protocol to study mouse V1 the authors provide new information by analyzing the strength of responses and orientation/direction tuning metrics at different depths from layers 1 to 4. The results appear to be robust and indicate that the percentage of cells that respond to the drifting gratings is highest in layer 4 and decreases as the imaging plane is moved superficially. This is accompanied by a decrease in response strength and an increase in orientation selectivity. The paper is well written and the data is clearly described, we have only minor queries and we feel the paper will provide useful information for our understanding and modelling of mouse V1.

Comments:

1) The authors mention cells that show a decrease in fluorescence upon visual stimulation and that they shifted the response of these cells by their minimum to remove negative values. It would be useful to get an impression of how many cells show these negative responses and whether they are more predominant in particular layers. Are the responses consistent with a general negative offset to all visual stimuli or does it look like an ‘inverted’ orientation tuning? A suggestion is to rectify the responses above 0 rather than shift. We have the impression that shifting may be wrong.

2) The paper could note other dependencies on depth, including Ringach et al (2002) in macaque. In addition, Van Hooser et al. 2013 noted a strong dependence of orientation selectivity on depth in the tree shrew. Finally, the differences between modulation index and 1-circular variance that were noted (and the difficulties with the modulation index) were explored in simulations by Mazurek et al. 2014.

3) The authors report statistics that are based on averages across all the cells at a particular depth plane. This is fine, but obviously throws away a lot of the interesting variability between cells. The authors might want to consider the use of linear mixed effects models which can account for the fact that the data is hierarchical and that cells are nested within mice. This may allow a more sophisticated analysis of the effect of depth on OSI and can also be extended to binomial data such as whether a cell is tuned or not. To restate, the statistics as performed by the authors are valid and correct, but there may be more in the data that can be revealed by more sophisticated approaches.

4)For measures such as OMI and DMI, which involve using the data to determine the preferred orientation/direction, it would be useful to test a cross-validated measure of this statistic. For example, select the preferred orientation/direction with one half of the trials and then calculate the statistic with the other half.

## Author Response

We thank the reviewers and the editor for their comments and suggestions to improve our manuscript. New text in the manuscript is highlighted in red font. Below we address the individual points.

1) “The authors mention cells that show a decrease in fluorescence upon visual stimulation and that they shifted the response of these cells by their minimum to remove negative values. It would be useful to get an impression of how many cells show these negative responses and whether they are more predominant in particular layers. Are the responses consistent with a general negative offset to all visual stimuli or does it look like an ‘inverted’ orientation tuning? A suggestion is to rectify the responses above 0 rather than shift. We have the impression that shifting may be wrong.”

The interpretation of negative signals with calcium imaging may be difficult. Because spontaneous firing rates in mouse V1 are low, it is unlikely that negative responses are caused by inhibition. There may be small negative signals due to noise around baseline for conditions in which neurons are not responding. However, the larger negative responses we are mostly concerned with are most likely due to shadowing from surface artery dilation (see Shen et al. 2012 Fig. 3). Given that surface vessels are untuned for stimulus orientation (see O'Herron et al. 2016 Fig. 1), we feel the most appropriate correction is to shift all of the responses by the most negative response-but see paragraphs below for new controls that we performed. Our shifting of responses by the most negative response assumes the true response for this condition is zero and the negative amplitude is due to shadowing. Because of the lack of tuning in the vessel dilation response, the shadowing effect should be equal for all stimulus conditions.

We had mentioned in the Materials & Methods that we fit the tuning curves without this correction and saw very similar results. We now have also added a new method following the reviewer's suggestion to rectify the responses rather than shifting. We simply made all negative responses zero and left the positive responses untouched. With the new correction, some of the previous positively-shifted responses will now be zero, leading to slightly higher selectivity values. So the overall values of the metrics do change slightly with the two different corrections used for negative responses. However, the significance of our paper relates to how these metrics (such as OSI) vary with neuronal depth and response strength. For these parameters, as we illustrate below, the type of correction used makes little difference.

The percent of cells that respond in our population remains identical because the “shift correction” we used is performed after data have already been tested for significant responsiveness. With the reviewer-inspired correction, the response amplitude and selectivity all show the same trends with depth and response strength (see Author Response Figure 1 below) as for our original “shift correction” (see Figures 2 thru' 6 in the manuscript). We have added text to the Materials & Methods to explain these new analytical controls.

Regarding the specific questions about how many cells showed negative responses and their distribution across depth: a) To assess if there was a population of neurons with substantial negative responses that might indicate something other than shadowing (e.g., neuronal inhibition), we analyzed significantly-responding cells that had at least half of their responses negative. 10% of cells (512 of 5020 significant responders) had half of their responses negative (fluorescence in stimulus window lower than in baseline window). Looking at the percentage of these cells across depth, we found no significant effect (R2 = 0.06, P = 0.14, linear regression, n = 35 imaged regions in 7 mice; ANOVA with depth as factor; P = 0.09; see Author Response Figure 2A below). b) We also plotted the tuning curves of the cells showing half of their responses negative. We aligned the responses by the preferred orientation (as was done in Figure 4 of the manuscript) and averaged across depths. As the new figure shows (Author response Figure 2B below), these responses have the same overall shape as with the “shift correction” (Figure 6 in the manuscript) but with a negative shift of the non-preferred orientations below 0

Author response Figure 1. “Correcting” negative responses by rectifying negative responses to zero show identical trends as the original correction described in the manuscript. A. Maximum response amplitude still increase with depth when using the new correction. B-E. Repeating the various metrics in the paper with the new correction, we still see the depth trends for the whole population and that there is a greater depth effect for the strongest third of responders. F. The population tuning curves at each depth show similar profiles as the original data in Figure 6 in the manuscript.

Author Response Figure 2. Properties of cells showing negative responses. A. The percentage of cells in each run in which at least half of the responses were negative. Conventions are as in main paper. B. Population tuning curves at each depth for the negative responding cells. Conventions as in Figures 4 and 6 in the manuscript.

We next compared the amplitude of the most negative response to that of the most positive response. If a cell was showing predominantly inhibition, the most negative response should have a greater absolute value than the peak positive response. The median (max negative response)/(max positive response) across the population was around 34%. Only 62 total cells showed a negative response with greater amplitude than the peak positive response. There was also no significant effect of depth on the negative/positive response ratio (ANOVA with 512 cells, depth and animal as factors: depth P=0.15; animal P=0.21; interaction P=0.12).

2) “The paper could note other dependencies on depth, including Ringach et al (2002) in macaque. In addition, Van Hooser et al. 2013 noted a strong dependence of orientation selectivity on depth in the tree shrew. Finally, the differences between modulation index and 1-circular variance that were noted (and the difficulties with the modulation index) were explored in simulations by Mazurek et al. 2014.”

We thank the reviewers for bringing the relevance of these papers to our attention. We had primarily focused on rodent studies since our data is from rodent, but we agree that previous work on these depth dependent effects in other species is important to acknowledge. So, we have added new text to the Discussion section of our manuscript to emphasize that studies in mice have not demonstrated these effects but they have been found in other species. We have also included the suggested citation regarding the merits of different tuning metrics.

3) “The authors report statistics that are based on averages across all the cells at a particular depth plane. This is fine, but obviously throws away a lot of the interesting variability between cells. The authors might want to consider the use of linear mixed effects models which can account for the fact that the data is hierarchical and that cells are nested within mice. This may allow a more sophisticated analysis of the effect of depth on OSI and can also be extended to binomial data such as whether a cell is tuned or not. To restate, the statistics as performed by the authors are valid and correct, but there may be more in the data that can be revealed by more sophisticated approaches.”

We agree with the reviewers that more analysis could potentially uncover other novel findings. Without more detail it is not entirely clear what parameters the reviewers were thinking of seeing but we hope to build on this work in the future with more complex stimuli and statistics. However, given that the reviewers did not have any problem with our analyses as performed and that the authors are currently scattered across multiple institutions, we hope that the current findings are sufficient for this publication. We would also like to emphasize that the depth trends are seen in each mouse and so this minimizes any concern that pooling cells within a depth plane might hide the strength of the trends. Performing statistics on populations of individual cells rather than on means across runs will likely lead to even higher statistical significance.

4) “For measures such as OMI and DMI, which involve using the data to determine the preferred orientation/direction, it would be useful to test a cross-validated measure of this statistic. For example, select the preferred orientation/direction with one half of the trials and then calculate the statistic with the other half.”

We thank the reviewers for this suggestion. This seems to stem from a concern that the metrics may not be completely accurate since the same data are used to first choose the preferred and non-preferred orientations and then to compute the metric. For the OMI, indeed the preferred stimulus is determined using the same data that the metric is computed from. For the DMI, this was not the case since the preferred and null directions were based on the peaks of the tuning curves fit to the data. Therefore, to assuage this concern, we recomputed the OMI using half the trials after determining the preferred orientation on the other half of the trials. As Author Response Figure 3 below shows, the trends across depth and response strength are quite similar to the original data (Figure 5 of the manuscript).

Author Response Figure 3. Cortical depth dependence of the Orientation Modulation Index (OMI) computed on half the trials. A. Average of OMI values for each mouse and imaging depth. B. Average grouped by neuronal response amplitude. Conventions as in figures in main paper.
